# Anthrax

**Published:** 1889-10

**Authors:** W. M. Barritt

**Affiliations:** Onarga, Ill.


### ANTHRAX.*

BY W. M. BARRITT, M. D., ONARGA, ILL.

I will not speak at length on the causes, pathology or symptoms of anthrax. I will say, however, that whatever peculiar constitutional vice, functional derangement, or specific cause gives

*Read at the thirty-ninth annual meeting of the Illinois State Medical Society, May 23d, 1889.

rise to this manifestation, pathologists have not been able to clearly demonstrate. That it is entirely local in its origin, without some predisposing influence, seems improbable. Its frequent occurrence in diabetics has suggested a line of research, but so many exceptions to the rule have prevailed, that not to the glycogenic function of the liver alone must we look for a solution.

Of the few cases that have come within my personal knowledge none have shown a condition worthy the dignity of the name, glycosuria. Traces of sugar were found in one case, but not in quantity sufficient to justify more than a probability that it was due to reflex origin.

Without entering into a discussion of the many theoretical questions with which our knowledge of the etiology of anthrax is involved, it is probably more practical to discuss some plan on which to base a rational system of managing the disease.

Whether due directly to inflammation of bundles of cellular tissue contained in the areolæ of the derma, as believed by Dupuytren to exist; whether an inflammation of the processes of cellular tissue which accompany the vessels and nerves through the true skin, as believed by Sanson ; whether Nelaton's view, that it was simply a product of secretion, or whether the more probable explanation of Dr. Warren that the existence of columns of adipose tissue leading from the panulus adiposus up to the roots of the lanugo hairs, taking an oblique direction in a line with the erectores pilorum, we have an inflammation of the adipose tissue resulting in suppuration of subcutaneous adipose tissue, which must either form an abscess or become diffuse.

The characteristic symptoms that are objectively presented are a hard, or boggy, dark or livid red swelling, convex, circumscribed, spreading slowly and attended with smarting or burning pain. On its most prominent or central part, numerous ulcerated points appear which give exit to a thin starch-like fluid—a kind of disintegration of skin takes place uniting the ulcerated points or fistulæ, forming a considerable opening from which a slough of cellular tissue is protruded and also through which pus constantly escapes. The tumor may vary in size from that of a half dollar to twelve or more inches in diameter.

In the treatment of anthrax but slow advancement has been made. Nearly a half century ago Dr. Robert Druitt wrote that "the objects of local treatment are to afford a free exit to sloughs and discharge, and to excite the diseased tissues to healthy suppuration and granulation." He says: "In the first place, therefore, a free incision should at once be made completely through the tumor, and if the tumor is extensive, it should be scored across by a second incision at right angle to the first."

Thus far the management of these cases by almost all surgeons has not materially changed down to the present time. Death not infrequently results from septicæmia, exhaustion, hemorrhage or embolism. A better knowledge of the value of antiseptics has contributed something to the treatment. Permanganate of potash has been spoken of with much confidence by some surgeons, who believe that anthrax may be materially abridged in duration, if not aborted, by the use of this remedy.

In a paper read by Professor Verneuil, of Paris, before the Academy of Medicine last year, carbolized spray was recommended; his plan is to spray the tumor for half an hour three or four times daily with a two per cent. solution of carbolic acid. Some surgeons, among whom are Guyon and Sereins, recommended injections of iodine solutions into and around the tumor. Parenchymetous injections of carbolic acid have been used with the result of a loss of about one case in from six to ten.

Dr. Collins, of Philadelphia, is reported to have had good results from curetting after first anæsthetizing and making a free crucial incision.

Mr. Herbert Page, Mr. Edmund Owen, and Mr. Rushton Parker reported to the *British Medical Journal* last year cases treated as above.

I want to present to you a plan of treatment that in my hands has proved quite successful. It consists in the use of peroxide of hydrogen freely injected into the tumor. Now, while the use of this agent has attained some popularity in the treatment of abscesses, its use has not, to my knowledge, been especially urged in the treatment of this painful and dangerous disease. Peroxide of hydrogen, now quite well known by name, is a solution of oxygen in water, and as ordinarily sent out by wholesale druggists, contains fifteen volumes of oxygen. It has a chemical formula of $H_2 O_2$. Among its important actions, it is antiseptic, disinfectant and germicide. When brought in contact with pus, a chemical reaction accompanied with effervescence occurs, which change completely destroys the pus and leaves the tissues in the best condition for healing. In anthrax the pus is so infiltrated through the tissues that even extensive excisions will not evacuate it, and even the curetting treatment above mentioned cannot reach all the ramifications of minute canals in which it has burrowed.

I first used peroxide of hydrogen for anthrax in November, 1886. The tumor was an enormously large one, measuring twelve by sixteen inches in diameter. I had incised it and had used freely a six per cent. solution of carbolic acid, had injected it hypodermically, and had even used the solution instead of water for mixing poultices applied. I had also used iodine and iodoform, but everything failed to arrest the progress of the disease until I began to use peroxide of hydrogen, and although septicæmia had exhibited itself by the production of several large secondary abscesses, the wound immediately presented a more healthy appearance; the blackened skin in a few hours assumed an arterial hue and united to the tissues below by first intention,

My plan of using peroxide of hydrogen is to inject it into the tumor hypodermically as far out as the margin, one or more times daily, injecting it freely also into the fistulous openings, finally dressing the wound with antiseptic cotton and iodoform, making a little pressure by the dressing.

If the theory that anthrax is in any sense a sequel of glycosuria has any foundation, no more potent remedy could be suggested than peroxide of hydrogen. Dr. Richardson, of London, and others, among whom was Dr. John Day, gave the most flattering reports of its use in the treatment of glycosuria. The treatment of anthrax by this agent is mild and soothing to pain, and avoids the danger and risks of anæsthesia in scraping. It routs the pus thoroughly, and at the same time destroys it, The treatment is adapted to all stages of the disease.

